# Mitochondrial DNA Structure in *Trypanosoma cruzi*

**DOI:** 10.3390/pathogens14010073

**Published:** 2025-01-14

**Authors:** Alfonso Herreros-Cabello, Francisco Callejas-Hernández, Manuel Fresno, Núria Gironès

**Affiliations:** 1 Centro de Biología Molecular Severo Ochoa, Consejo Superior de Investigaciones Científicas, Universidad Autónoma de Madrid, Cantoblanco, 28049 Madrid, Spain; 2 Bloomberg School of Public Health, Johns Hopkins University, Baltimore, MD 21205, USA; 3 Instituto Sanitario de Investigación Princesa, 28006 Madrid, Spain

**Keywords:** *Trypanosoma cruzi*, kinetoplast, maxicircles, minicircles

## Abstract

Kinetoplastids display a single, large mitochondrion per cell, with their mitochondrial DNA referred to as the kinetoplast. This kinetoplast is a network of concatenated circular molecules comprising a maxicircle (20–64 kb) and up to thousands of minicircles varying in size depending on the species (0.5–10 kb). In *Trypanosoma cruzi*, maxicircles contain typical mitochondrial genes found in other eukaryotes. They consist of coding and divergent/variable regions, complicating their assembly due to repetitive elements. However, next-generation sequencing (NGS) methods have resolved these issues, enabling the complete sequencing of maxicircles from different strains. Furthermore, several insertions and deletions in the maxicircle sequences have been identified among strains, affecting specific genes. Unique to kinetoplastids, minicircles play a crucial role in a particular U-insertion/deletion RNA editing system by encoding guide RNAs (gRNAs). These gRNAs are essential for editing and maturing maxicircle mRNAs. In *Trypanosoma cruzi*, although only a few studies have utilized NGS methods to date, the structure of these molecules suggests a classification into four main groups of minicircles. This classification is based on their size and the number of highly conserved regions (mHCRs) and hypervariable regions (mHVRs).

## 1. Introduction to the *Trypanosoma cruzi* Kinetoplast

*Trypanosoma cruzi* (*T. cruzi*) is a unicellular protist responsible for Chagas disease, also known as American Trypanosomiasis. This chronic disease is endemic to Latin America and is classified as a neglected tropical disease [1]. The life cycle of *T. cruzi* is quite intricate, involving a blood-feeding triatomine insect vector and various mammalian hosts. This parasite undergoes several biological stages. In the midgut of the triatomine, non-infective epimastigotes transform into infective metacyclic trypomastigotes. Once inside a host, these trypomastigotes invade cells and differentiate into replicative amastigotes, which later become infective trypomastigotes that enter the bloodstream [2].

*T. cruzi* exhibits a huge genomic diversity and plasticity, considering both nuclear and mitochondrial genomes [3]. This genetic variability has led to the development of numerous classification methods for the many strains identified. These classifications are based on conserved genetic sequences, including genomic, mitochondrial, and microsatellite DNA. Nowadays, six discrete typing units (DTUs), known as TcI-VI, and a seventh one called TcBat, are the principal method for strain typing. DTUs are defined as “sets of stocks that are genetically more related to each other than to any other stock and that are identifiable by common genetic, molecular or immunological markers” [4,5]. Moreover, researchers have proposed that the genetic variability of the parasite may contribute to the different clinical manifestations of Chagas disease [6,7,8].

Kinetoplastids display a single large mitochondrion per cell, and their DNA is constituted by a network of concatenated circular molecules known as the kinetoplast or kDNA (or mtDNA), including several maxicircles (20–64 kb) and thousands of minicircles (0.5–10 kb) of different sizes depending on the specie [9,10,11]. Interestingly, many essential cellular processes take place in the mitochondria, such as oxidative phosphorylation, which is linked to ATP production. This process is carried out by a series of protein complexes in the electron transport chain (ETC), known as complexes I-IV (cI-cIV), and ATP synthase [12]. In trypanosomatids, cII consists entirely of subunits encoded by nuclear DNA. However, complexes I, III, IV, and ATP synthase include at least one subunit encoded by the mitochondrial genome [13].

According to different studies, the metacyclogenesis process in *T. cruzi* involves significant morphological changes affecting cellular structures, such as the DNA configuration within the kinetoplast. In the later stages during the metacyclogenesis, the kinetoplast transitions from a densely packed structure to a more globular and less dense form. Subsequently, the kinetoplast shifts from the anterior end (epimastigotes) to the posterior region of the cell body (metacyclic trypomastigotes) [14]. Furthermore, during the posterior amastigogenesis, after the host-cell invasion, parasites undergo a folding process, bringing their posterior end closer to their anterior end. This folding facilitates the repositioning of the basal body and kinetoplast from this posterior region, typical of trypomastigotes, to the anterior region again, which is characteristic of the amastigote stage [15].

Notably, the topological state of the kinetoplast is regulated by topoisomerases, which are responsible for catenating and decatenating the circular DNA molecules. Topoisomerase II is not expressed in metacyclic trypomastigotes, although its mRNA is present across all stages. This suggests that its mRNA might be stored in a translation-repressed form in trypomastigotes. Also, kinetoplast-associated proteins play a crucial role in the organization of the kinetoplast, and their distribution varies depending on the stage of the parasite [14].

The mitochondrial genome complexity is added to a unique kDNA S-phase in addition to the traditional G1, S, and G2/M stages of the cell cycle. However, many molecular players involved in the later stages, particularly those responsible for kDNA division and segregation, remain unidentified. Recently, a study investigated the role of Aurora kinase genes in *T. cruzi* (TcAUK1, TcAUK2, and TcAUK3) that are involved in the cell cycle. Detailed analysis of TcAUK1 localization revealed that this protein behaves like a canonical chromosome passenger protein, associated with the mitotic spindle during nuclear division. Additionally, during interphase, TcAUK1 is located on both sides of the kinetoplast, and its overexpression in epimastigotes results in a delayed G2-M transition, likely due to its impact on the initiation of kinetoplast duplication [16]. In *Leishmania major*, a single Aurora gene (Lmairk) has been reported [17]; however, its function has not yet been studied. Meanwhile, in *Trypanosoma brucei*, three Aurora genes were identified, but gene-silencing experiments revealed that only TbAUK1 is functional [18].

## 2. Maxicircle Diversity and Composition

Kinetoplastid maxicircles contain mitochondrial genes similar to those found in other eukaryotes [19]. Their sequences are divided into two primary regions: a coding region (CR), which is highly conserved among different species [20,21,22], and a divergent or variable region (DR), which is challenging to sequence due to its repetitive nature and variability in length [23].

Recent advancements in third-generation sequencing technologies have uncovered that the length of maxicircle sequences can vary significantly among trypanosomatid species [23]. Short-length sequencing technologies (such as Illumina, and including paired-end) often fall short in assembling the complete maxicircle in *T. cruzi*, leading most studies to focus solely on the coding regions with short flanking sequences [24,25]. Repetitive elements, such as tandem repeats and large duplications, complicate the assembly process and can introduce errors even in coding sequences. However, long-read sequencing technologies, such as PacBio, can mitigate these challenges [26].

The first *T. cruzi* maxicircles were described in 2006 [27] through a comparative analysis of the CL-Brener and Esmeraldo strains. The maxicircle sizes were estimated to be 22 kb and 28 kb, respectively, but a collapsed zone of repetitive sequences hindered their complete assembly. After this analysis, in 2011, the maxicircle coding region (CR) of the SylvioX10 strain was sequenced by PCR amplification of specific fragments, followed by assembly and comparison with the CL-Brener and Esmeraldo maxicircles [28], confirming the synteny of these CRs among different *T. cruzi* clades.

Later, in 2020, Gerasimov et al. utilized PacBio reads to assemble 17 maxicircles of different trypanosomatids, including two strains of *T. cruzi* (Dm28c and TCC), thereby expanding the known collection of divergent region (DR) structures and solving the problems with their repetitive regions [23]. On one hand, the Dm28c maxicircle was the longest, considering all the species (including *Leishmania* and *T. brucei*), with 47.39 kb, and both Dm28c and TCC maxicircles displayed the largest DR, even doubling the size of the other maxicircles. On the other hand, the CRs of all assembled maxicircles were comparable in length and demonstrated a high degree of synteny. Furthermore, in this study, DR organization was also analyzed by identifying two distinct regions, the P5 and P12 elements, based on their proximity to the *nd5* and *12S* genes, respectively. Similar structures have previously been reported in *Trypanosoma lewisi* [29]. The P12 element comprises short, highly repetitive units organized in repeat arrays of varying length and that are very dynamic [30,31], whereas the P5 element is a tandem repeat with a large period, but it has a small number of repetitions. This architecture appears to be a typical feature of the DRs in trypanosomatid maxicircles.

Subsequently, two articles published in 2021 sequenced new maxicircles, significantly advancing our understanding in this field. In the first one, the authors assembled the maxicircles of Y and Bug2148 [32], displaying minimal sequence-length variations in the CRs and supporting the notion that the well-documented differences between strains in the maxicircle are primarily due to the DRs, consistent with the findings of Gerasimov et al. Additionally, for the first time in *T. cruzi*, the researchers observed differences in %GC content between the CR and DR; hence, the DR is highly enriched in AT nucleotides (up to 76%), whereas the CR exhibits a more balanced nucleotide composition (approximately 47.6% GC content).

**Figure 1:**
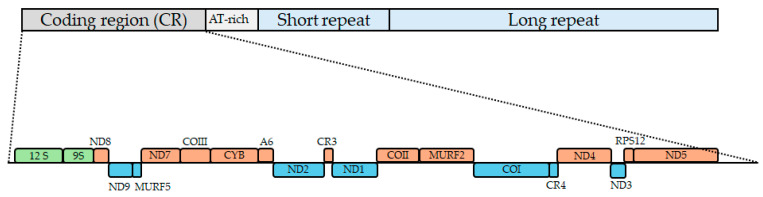
**The architecture of the *T. cruzi* maxicircle.** The coding region (CR), AT-rich, short-repeat region, and long-repeat region are shown, according to Berná et al., [33] as well as the genes of the CR: green for the ribosomal genes, blue for the genes in the negative strand, and brown for the genes in the positive strand.

In the second manuscript from 2021, Berná et al. described high-quality assemblies of maxicircle genomes from the six DTUs of *T. cruzi*, including the resolution of repetitive regions, using long reads from PacBio and Nanopore and post-corrected with Illumina reads [33]. They reassembled the maxicircle of Dm28c and TCC, increasing the DR size in 2–3 kb compared to Gerasimov et al. [23]. Notably, these researchers separated the DR into three different sections: the short repeats (SRs) and long repeats (LRs) and the AT-rich region (AT content 83%). This last one presents a size < 1 kb and is located among the CR and the SR (Figure 1). This fits with the previous observations of Callejas-Hernández et al. and Gerasimov et al. in other *T. cruzi* strains. Regarding the characteristics of these regions, differences were found among DTUs in the length of the SR (from ~2.1 kb to ~6.8 kb), LR (from ~14.3 kb to ~30.3 kb), and the AT-rich region (from ~0.1 kb to ~1 kb).

Table 1 summarizes all these assembled maxicircles of *T. cruzi* by strains, showing the lengths of both CRs and DRs. Interestingly, the Y strain maxicircle was sequenced twice obtaining different results in the length of both CR and DR, which raises questions about whether they are the same strain or the impact of its maintenance under laboratory conditions between distinct research groups may have on this strain. Also, in the case of the Bug2148 strain, the sequence length is the largest recorded to date at 64.11 kb, indicating a potential unknown level of complexity in some *T. cruzi* strains. The DTU and the origin of the Bug2148 strain are currently under debate. Phylogenetic analysis using the complete coding regions of maxicircles associates it with TcI, although it has been previously classified as TcV [33].

Altogether, these genomic studies have been used to establish the complete repertoire of the 20 genes of the *T. cruzi* maxicircle (Figure 1). Among them, 12S rRNA and 9S rRNA encode ribosomal RNA components essential for mitochondrial protein synthesis. COI, COII, and COIII encode subunits of cytochrome c oxidase, which is involved in the electron transport chain and ATP production. The Cyb gene encodes cytochrome b, a component of the bc1 complex in the electron transport chain. Also, there are several genes (ND1, 2, 3, 4, 5, 7, 8, and 9) that encode the NADH dehydrogenase subunits of the electron transport chain, while CR3 and CR4 are cytosine-rich genes that are believed to encode homologous proteins to ND4L and ND6. Finally, RSP12 is the ribosomal protein S12, A6 is the ATPase 6 and the MURFs (Maxicircle Unidentified Reading Frames) genes encode proteins of unknown function, but they could be involved in mitochondrial processes.

However, some modifications have been described for different strains as insertions/deletions of specific genes or sequences. These changes may be common to a given lineage or exclusive of a particular strain. According to Berná et al., the Y strain (TcII) exhibits two deletions of 452 bp and 1071 bp. The first deletion disrupts the *nd7* gene, while the second causes a 5′ deletion of *nd2*, the complete elimination of *cr3*, and a 3′ deletion of *nd1*, leaving only the first 167 nucleotides at the 5′ end [33]. Interestingly, in strains isolated from asymptomatic patients, the *nd7* truncation was also detected [34], demonstrating that it is not exclusive to TcII. While this deletion is not found in either the Esmeraldo or Berenice strains, both strains show a similar deletion impacting the *nd4* gene [27].

In contrast, the BolFc10A strain (TcV) has an interruption of the *nd2* gene, and it contains two insertions of 1408 bp and 1017 bp, separated by 893 bp, belonging to minicircle sequences [33]. The 1408 bp insertion corresponds to an entire minicircle with four conserved regions disrupting the *nd4* gene, while the 1017 bp insertion is a fragment of a minicircle sequence situated in the intergenic region between the *nd4* and *nd3* genes. Despite being different minicircles, both insertions carry the same gRNA for the *nd3* gene. Moreover, a predicted gRNA for the *murf2* gene was found in the SylvioX10 maxicircle between the *nd4* and *cr4* genes, and it was also confirmed in CL Brener, Y, and Bug2148 strains [28,32].

Therefore, all of these modifications affecting, above all, mitochondrial *nd* genes (that encode for NADH dehydrogenase subunits) generate a debate about the existence of a functional complex I in *T. cruzi* [35]. In this parasite, succinate serves as the primary electron donor for the ETC, rather than NADH. Respiration is inhibited by malonate, a competitive inhibitor of mitochondrial succinate dehydrogenase, rather than by complex I inhibitors [36]. Further analysis involving mitochondrial respiration, with distinct strains of *T. cruzi*, is mandatory to clarify the role of the maxicircle genes and the functionality of complex I.

## 3. Minicircles Diversity and Composition

Minicircles are unique to kinetoplastids and play a key role in a U-insertion/deletion RNA-editing system by encoding guide RNAs (gRNAs) [37,38,39]. This process is critical for editing the maxicircles mRNAs and their maturation. Regarding its length and composition in *T. cruzi*, since the 1980s, several studies have shown that the set of minicircles had a conserved structure across strains, approximately 1.4 kb in size. These minicircles were organized into four highly conserved regions (mHCRs) of 120 bp, positioned 90 degrees apart, and the same number of 330 bp hypervariable regions (mHVRs) interspersed among the conserved regions [40,41,42,43,44,45]. The diversity of mHVRs has garnered attention; hence, these regions encode the gRNAs that guide the editing of many mitochondrial mRNAs, transforming these primary transcripts into functional messages [11,46].

Furthermore, minicircles contain three conserved sequence blocks (CSBs). CSB-1 (10 bp) and CSB-2 (8 bp) exhibit lower homology between species, while CSB-3 (12 bp), known as the Universal Minicircle Sequence (UMS), is conserved across most kinetoplastids and serves as the origin of minicircle replication [47]. CSBs number, location, and size vary between species [43,48,49,50]. Specifically, CSB-3, or UMS, is the binding site for the UMS binding protein (UMSBP), associated with minicircle replication and kDNA segregation [51]. Although this specific protein has been widely studied in other species, such as *Crithidia fasciculata* [52,53], its presence was confirmed in *T. cruzi* by a proteomic study [54]. According to all of the results available, the consensus sequences for each CSB, including *T. cruzi*, would be as follows: CSB-1: AGGGGCGTTC; CSB-2: CCCCGTAC; and CSB-3: GGGGTTGGTGTA.

However, two recent studies have changed all of these statements about the minicircles of *T. cruzi*. In the first one, in 2021, the complete repertoire of minicircles of the Y strain was published [32], detecting 286 different minicircles. Contrary to the previous affirmations, Y strain minicircles displayed four distinct groups of size: 42 minicircles between 336 and 376 bp (group 1), 9 minicircles between 698 and 769 bp (group 2), 47 minicircles between 1051 and 1095 bp (group 3) and, finally, the largest group consisted of 188 minicircles between 1357 and 1448 bp (group 4). Thus, the authors proposed a new classification into four groups based on size ranges, with each group separated by approximately 300 bp. Interestingly, these minicircle subtypes had already been documented and suggested in the 1980s [55,56]. Furthermore, analyzing the number and structure of the mHCRs and mHVRs, the authors discovered an increasing number of both regions according to the minicircle size: from one mHCR and one mHVR in the group 1 minicircles, to four mHCRs and four mHVRs in the group 4 minicircles. In addition, in the mHCRs of the Y-strain minicircles, the composition and number of the CSB-1, CSB-2, and CSB-3 were also analyzed. The authors highlighted that the theoretical consensus sequences were larger than the previous ones for *T. cruzi*, featuring more conserved nucleotides at both ends in all the CSBs. Interestingly, small differences, such as changes in the nucleotide proportion in a position or nucleotide insertions, between mHCRs of distinct minicircle groups and between the mHCRs of the same minicircle group were also detected.

**Figure 2:**
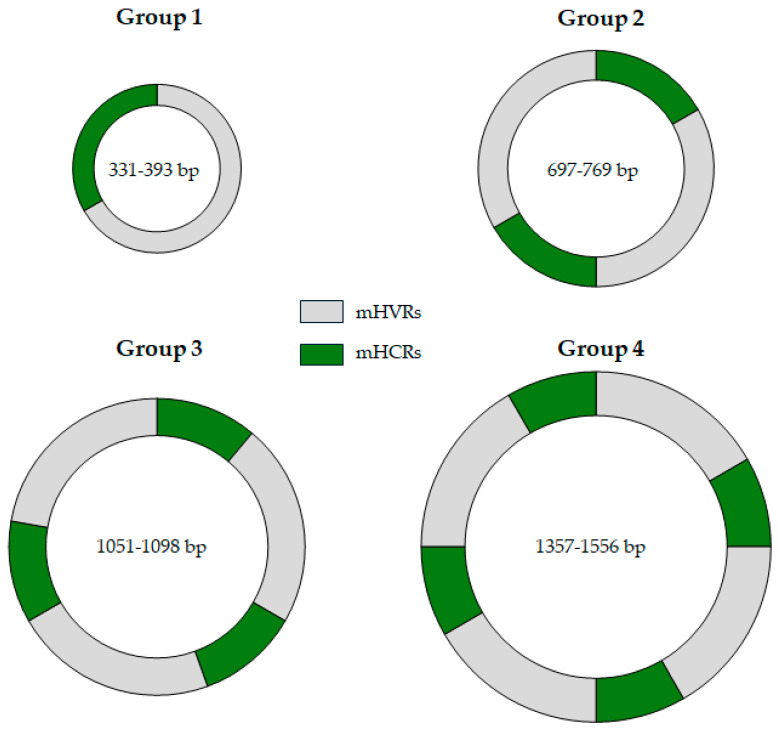
**Architecture of the minicircles of TcI and TcII strains of *T. cruzi*.** Minicircle groups 1–4 of sizes are shown, along with their ranges, according to the data of Callejas-Hernández et al. [32] and Gómez-Palacio et al. [57]. mHCRs: highly conserved regions (green). mHVRs: hypervariable regions (grey). bp = base pairs.

In the second one, in 2024, this minicircle organization was confirmed, in which the authors established the minicirculome of 50 clones of TcI populations [57]. They identified a total of 8254 minicircles, with each clone having an average of 165 minicircles. They categorized these minicircles into four distinct groups: group 1 (331–393 bp), group 2 (697–751 bp), group 3 (1059–1098 bp), and group 4 (1409–1556 bp), ranges very similar to the previous research with the Y strain. Table 2 and Figure 2 compile all of these minicircle groups’ data. The primary difference is observed in group 4, where TcI clones exhibit a slightly larger size compared to the Y strain (TcII).

A first study with the minicircle sequences of the CL-Brener and Esmeraldo strains described 108 potential gRNAs from 248 minicircle mHVRs, and two gRNAs within CL-Brener maxicircle [11]. After that, in 2021, Rusman et al. identified a total of 7,476,003 gRNAs across nine *T. cruzi* strains belonging to the six DTUs [46]. Subsequently, the gRNAs were clustered into gRNA classes based on the editing region and sequence similarity. A total of 1334 gRNA classes were identified, and their number per strain varied, ranging from 104 to 401. Notably, TcV strains had up to three- or four-times fewer gRNA classes than other DTUs. Altogether, it suggests rapid changes in gRNA repertoires and potential gRNA redundancy.

Remarkably, Gómez-Palacio et al. identified a total of 9849 gRNAs in the minicircles of TcI clones specifically [57]. These gRNAs were predicted for 16 out of 18 tested genes, with no gRNAs found for the *nd2* gene in any of the analyzed clones, and only 1 gRNA identified for the *nd7* gene. Moreover, significant differences were observed in the number of predicted gRNAs and the abundance of edited genes among strains. For the most prevalent minicircle group (group 4), a total of 8562 gRNAs were predicted for all edited genes, except *nd2*. In contrast, group 1 had 802 gRNAs, group 2 had 666, and group 3 had 1135. No predicted gRNAs were found for the *nd8* and *nd9* genes in group 1; the *nd9* and *cr4* genes in group 2; and the *nd1*, *nd9*, and *cr4* genes in group 3.

Compared with different *Trypanosoma* species, *T. cruzi* possesses four groups of minicircles, whereas *T. rangeli* displays three distinct groups of minicircle sequences, termed KP1, KP2, and KP3, based on the number of conserved regions. These classes include one conserved region (KP1), two conserved regions positioned 180 degrees apart (KP2), or four conserved regions located at 90-degree intervals (KP3) [58,59]. Moreover, in *T. cruzi*, minicircle sizes range from 336 to 1448 bp, while *T. rangeli* shows a more consistent size of 1.6–1.8 kb [60]. Also, *Trypanosoma copemani* minicircles are approximately 2048 bp and are categorized into two groups based on the number of conserved and variable regions: G1M1 minicircles (two conserved and two variable regions) and G1M2 minicircles (four conserved and four variable regions) [50]. In *Leishmania*, *Leishmania major* has 97 different minicircles, whereas *Leishmania infantum* has only 49. Despite this, the sizes of their minicircles are quite uniform, ranging from 660 to 876 bp in *L. major* and from 775 to 832 bp in *L. infantum* [22]. Additionally, *Leishmania* minicircles contain only one conserved region, in contrast to the *T. cruzi* model.

## 4. Conclusions

The kinetoplast, or kDNA, is a network of concatenated circular molecules in kinetoplastids. It consists of a maxicircle (20–64 kb) and thousands of minicircles (0.5–10 kb), with variable sizes among species. In *T. cruzi*, NGS techniques have enabled the complete sequencing of maxicircles from different strains over the past decade. This research has confirmed the CR synteny and the DR diversity, which appears to be divided into three sections. These analyses have also revealed various modifications in maxicircle genes among strains, although much remains to be understood about their origins. Additionally, some aspects, such as the specific roles of certain maxicircle genes, particularly those encoding NADH dehydrogenase subunits, require further investigation.

Recently, there has been a significant shift in understanding the *T. cruzi* minicircles. Initially, it was thought that minicircles were of a single size (1.4 kb), with four mHCRs and four mHVRs. However, recent studies have shown that *T. cruzi* minicircles can be classified into four main size groups, each with an increasing number of mHCRs and mHVRs. This conclusion is based on studies of TcI and TcII strains, and further research is needed to confirm it as a general pattern among strains. However, many questions remain unanswered, such as the origin of these four different size structures of minicircles and the significant diversity of gRNAs found among strains.

## Figures and Tables

**Table 1:** **Information about the different complete, assembled maxicircles of *T. cruzi*.** CR: coding region. DR: divergent/variable region.

Strain	DTU	Length (kb)	CR (kb)	DR (kb)	GenBank Accession	Reference
**Dm28c**	I	50.48	15.36	35.12	MW421590	[33]
**Y**	II	38.79	13.85	24.94	MW421591	
**MT3663**	III	44.19	15.29	28.89	MW567142	
**JoseJulio**	IV	44.28	15.26	29.02	MW421592	
**BolFc10A**	V	34.80	17.54	17.27	MW567141	
**TCC**	VI	42.48	15.34	27.14	MW407947	
**Y**	II	24.09	15.18	8.91	MW732647	[32]
**Bug2148**	I/V	64.11	15.18	48.93	MW732648	

**Table 2:** **Group composition of the assembled minicircles of *T. cruzi*.** The numbers of mHCRs and mHVRs for each group and DTU are displayed, along with their percentage of the total, based on the data of Callejas-Hernández et al. [32] and Gómez-Palacio et al. [57].

Group	DTU	Size Range (bp)	Number of mHCRs	Number of mHVRs	% of Minicircles
**1**	TcI	331–393	1	1	12.13
	TcII	336–376	1	1	14.69
**2**	TcI	697–751	2	2	5.21
	TcII	698–769	2	2	3.15
**3**	TcI	1059–1098	3	3	10.17
	TcII	1051–1095	3	3	16.43
**4**	TcI	1409–1556	4	4	72.49
	TcII	1357–1448	4	4	65.73
